# The Inflammasome Signaling Pathway Is Actively Regulated and Related to Myocardial Damage in Coronary Thrombi from Patients with STEMI

**DOI:** 10.1155/2021/5525917

**Published:** 2021-05-27

**Authors:** Jostein Nordeng, Hossein Schandiz, Svein Solheim, Sissel Åkra, Pavel Hoffman, Borghild Roald, Bjørn Bendz, Harald Arnesen, Ragnhild Helseth, Ingebjørg Seljeflot

**Affiliations:** ^1^Center for Clinical Heart Research, Oslo University Hospital Ullevål, Norway; ^2^Department of Cardiology, Oslo University Hospital Ullevål, Norway; ^3^Faculty of Medicine, University of Oslo, Norway; ^4^Department of Pathology, Akershus University Hospital, Norway; ^5^Section for Interventional Cardiology, Department of Cardiology, Oslo University Hospital Ullevål, Norway; ^6^Department of Pathology, Oslo University Hospital Ullevål, Norway; ^7^Department of Cardiology, Oslo University Hospital Rikshospitalet, Norway

## Abstract

**Background:**

The Nod-Like-Receptor-Protein-3 (NLRP3) inflammasome and the Interleukin-6 (IL-6) pathways are central mechanisms of the inflammatory response in myocardial reperfusion injury. Expanding our knowledge about the inflammasome signaling axis is important to improve treatment options. In a cross-sectional study, we aimed to study presence, localization, and genetic expression of inflammasome- and IL-6- signaling-related proteins in coronary thrombi and circulating leukocytes from ST-elevation myocardial infarction (STEMI) patients, with relation to myocardial injury and time from symptoms to PCI.

**Methods:**

Intracoronary thrombi were aspirated from 33 STEMI patients. Blood samples were drawn. mRNA of Toll-Like-Receptor-4 (TLR4), NLRP3, caspase 1, Interleukin-1*β* (IL1-*β*), Interleukin-18 (IL-18), IL-6, IL-6-receptor (IL-6R), and glycoprotein 130 (gp130) were isolated from thrombi and circulating leukocytes and relatively quantified by RT-PCR. A part of each thrombus was embedded in paraffin for histology and immunohistochemistry analyses.

**Results:**

Genes encoding the 8 markers were present in 76-100% of thrombi. Expression of TLR4 in thrombi significantly correlated to troponin T (*r* = 0.455, *p* = 0.013), as did NLRP3 (*r* = 0.468, *p* = 0.024). Troponin T correlated with expression in circulating leukocytes of TLR4 (*r* = 0.438, *p* = 0.011), NLRP3 (*r* = 0.420, *p* = 0.0149), and IL-1*β* (*r* = 0.394, *p* = 0.023). IL-6R expression in thrombi correlated significantly to troponin T (*r* = 0.434, *p* = 0.019), whereas gp130 was inversely correlated (*r* = −0.398, *p* = 0.050). IL-6 in circulating leukocytes correlated inversely to troponin T (*r* = −0.421, *p* = 0.015). There were no significant correlations between genes expressed in thrombi and time from symptom to PCI.

**Conclusions:**

The inflammasome signaling pathway was actively regulated in coronary thrombi and in circulating leukocytes from patients with STEMI, in association with myocardial damage measured by troponin T. This supports the strategy of medically targeting this pathway in treating myocardial infarction and contributes to sort out optimal timing and targets for anti-inflammatory treatment. The study is registered at clinicaltrials.gov with identification number NCT02746822.

## 1. Introduction

Acute ST-elevation myocardial infarction (STEMI) is usually caused by plaque rupture and subsequent thrombus formation in a coronary artery. Restoring blood flow as soon as possible is of great importance in order to limit myocardial damage, and urgent revascularization by thrombolysis or acute percutaneous coronary intervention (PCI) is the cornerstone of treatment of these patients today [1]. In selected cases of large residual thrombus burden after the initial restoration of blood flow, thrombus aspiration is recommended [1].

Despite this treatment, acute coronary syndrome (ACS) remains one of the leading causes of death in the western world [2]. Considerable research has been performed to further reduce myocardial damage caused by coronary thrombi. A special focus has been on inflammation and immune-mediated damage, with the hypothesis that inflammatory responses might be excessive and harmful to myocardial tissue rescued by revascularization, i.e., myocardial reperfusion injury, and contribute substantially to the final size of the myocardial infarction (MI) [3]. However, anti-inflammatory interventions in clinical studies have mostly failed to reduce MI-size or improve patient outcome [4, 5]. This might be due to the great complexity of the inflammatory responses, with different players, that might play different roles during a myocardial infarction and the following repair phase. Some studies have reported changes in cellular composition [6–9], and our research group has previously shown genetic expression of inflammatory proteins in coronary thrombi in STEMI patients to be related to the time axis from symptoms to PCI [10]. Further knowledge about the molecular mechanisms involved is needed to sort out potential therapeutic options. Percutaneous thrombus aspiration provides an opportunity to study characteristics of the occluding thrombus *ex vivo*.

Two inflammatory signaling systems have been of particular interest, the Nod-Like-Receptor-Protein-3 (NLRP3) inflammasome and the interleukin-6 (IL-6) signaling system. The NLRP3 inflammasome regulates the activation of caspase 1, which induces pyroptosis and activates the central proinflammatory cytokines Interleukin-1*β* (IL-1*β*) and Interleukin-18 (IL-18) [11, 12]. The mechanisms behind the regulation and activation of the NLRP3 inflammasome are not fully understood, but the Toll-Like-Receptor-4 (TLR4) seems to be an important mediator [13]. The Cantos trial showed that canakunimab, a human monoclonal antibody blocking IL-1*β*, prevents recurrent cardiovascular events [14], indicating the NLRP3 inflammasome pathway to be of special importance.

IL-6 is involved in a broad specter of inflammation and infection responses [15]. In classic signaling, IL-6 binds to membrane-bound IL-6 receptor (IL-6R), which interacts with the signaling receptor protein glycoprotein 130 (gp130), mediating anti-inflammatory effects. In trans-signaling, IL-6 binds to a soluble form of IL-6R (sIL-6R), exerting an effect on membrane-bound gp130, mediating downstream proinflammatory responses [16]. Gp130 also exists in a soluble form, sgp130, which can bind to soluble IL-6/IL-6r-complex and thereby inhibit trans-signaling [17]. The IL-6R antagonist tocilizumab has been shown to reduce myocardial injury assessed by troponin T in patients with non-ST-elevation myocardial infarction (NSTEMI) [18]. The NLRP3-inflammasome and the IL-6 pathway seem to be linked by IL-1*β*-stimulating production of IL-6 [19]. Further downstream C-Reactive-Protein (CRP), produced in the liver as a response to IL-6, has been demonstrated to be a sustainable clinical marker of cardiovascular disease [19, 20].

We hypothesize that inflammasome- and IL-6-signaling-related proteins are present and actively regulated in coronary thrombi. We therefore aimed to investigate the genetic expression, presence, and localization of the inflammasome- and IL-6 signaling-related proteins in coronary thrombi from STEMI patients, and whether these were associated with the degree of myocardial injury and time from symptoms to PCI. Circulating levels and gene regulation in circulating leukocytes were also assessed.

## 2. Methods

### 2.1. Study Design and Population

33 patients admitted to Oslo University Hospital Ullevål with STEMI treated with primary PCI in the time period of August 2015 to January 2019 were included in the thrombus aspiration in ST-elevation myocardial infarction (TASTI) study. Inclusion criteria were patients of both sexes, age 18-85 years, with typical symptoms of acute myocardial infarction and STEMI in ECG, treated with primary PCI with thrombus aspiration. STEMI was defined as new significant ST-elevation in at least two adjacent ECG-leads. Significant ST-elevation was defined as ≥0.1 mV in the standard leads and precordial lead V4-V6, and ≥0.2 mV in precordial lead V1-V3. Presumed new left bundle branch block or typical posterior infarction pattern, with ST-depressions in lead V1-V4, was considered as STEMI equivalents. Exclusion criteria were signs of infection, pulmonary embolism, chronic obstructive pulmonary disease or arrhythmias, abnormal renal or liver function, autoimmune disease, and malignant disease. The study was approved by the Regional Committee of Medical Research Ethics in South-Eastern Norway with approval reference number 2015/169 and conducted according to the Declaration of Helsinki. All patients gave their written informed consent. The study is registered at clinicaltrials.gov with identification number NCT02746822.

Intracoronary thrombi were retrieved by standard aspiration catheter, washed with saline, and split in two. One part was stored in 10% buffered formalin, chemically processed, and paraffin-embedded for later histology and immunohistochemistry analyses. The other part was snap-frozen in RNA-later solution (Qiagen, Hilden, Germany) and kept frozen at -80°C for later isolation of RNA and gene expression analyses. Peripheral blood samples, including Pax-gene tubes (PreAnalytix, Hombrechtikon, Switzerland), were drawn at the end of the PCI procedure and repeated the next morning. Serum was processed by centrifugation at 2500 × g for 10 min and stored at -80°C until analysis. Demographic variables were recorded. Cardiac troponin T values (cTnT) were measured in serum at the hospital central laboratory by commercial electrochemiluminescence immunoassay (third-generation cTnT, Elecys 2010, Roche, Mannheim, Germany). The interassay coefficient of variability was 7%.

### 2.2. Laboratory Analyses

#### 2.2.1. Gene Expression Analyses

RNA was isolated from the snap-frozen part of the thrombi using High Pure RNA Tissue Kit with the addition of Proteinase K Solution (Roche Diagnostics GmbH, Mannheim, Germany), stabilized by lysing buffer, and homogenized by the use of termomixer (Termomixer Eppendorf, Eppendorf AG, Hamburg, Germany) and stainless steel grinding balls (Qiagen GmbH, Hilden, Germany). RNA from the PAXgene tubes was isolated by use of the PAXgene® Blood RNA Kit (PreAnalytix, Qiagen, GmBH), with an extra cleaning step (RNeasy®MinElute® Cleanup Kit, Qiagen). Quality and quantity of RNA (ng/*μ*L) were evaluated by use of the NanoDrop™ 1000 Spectrophotometer (Thermo Scientific, Wilmington, Delaware, USA).

cDNA was synthesised from an equal amount of RNA with qScript™ cDNA superMix (Quanta Biosciences, Gaithersburg, Maryland, USA). Real-time PCR was performed on a ViiA™ 7 instrument (Applied Biosystems, Foster City, CA, USA) using TaqMan® Universal PCR Master Mix (P/N 4324018) and the following TaqMan® assays: TLR4 (Hs00152939_m1), NLRP3 (Hs00918082_m1), Caspase-1 (Hs00354836_m1), IL-1*β* (Hs01555410_m1), IL-18 (Hs00155517_m1), IL-6 (Hs00174131_m1), IL-6R (Hs01075664_m1), and gp130 (Hs00174360_m1) (Applied Biosystems). *β*2-microglobulin (HS99999907_m1) (Applied Biosystems) was used as endogenous control, and mRNA levels were determined by relative quantification (RQ) using the Delta Delta C(T) Method (DDCT) [21]. With a relative quantification (RQ) value of 1.0, the gene analyzed was equally expressed as the reference sample.

#### 2.2.2. Circulating Levels

Commercially available ELISA kits were used for the determination of circulating levels of IL-6, IL-6R, gp130 (Quantikine® HS ELISA, R&D Systems®, Minneapolis, USA), and IL-18 (MBL, Medical & Biological Laboratories CO., LTD., Nagoya, Japan). Coefficients of variation in our laboratory were 10.6%, 3.6%, 5.2%, and 2.6%, respectively.

#### 2.2.3. Histology and Immunohistochemistry Analyses

The formalin-fixed, paraffin-embedded thrombi were serially sectioned at 3.5 *μ*m.  Section 1 and 7 were stained with hematoxylin and eosin (HE). The HE sections were morphologically staged into different thrombus age groups according to the criteria listed in Supplementary Table 1. These are based on criteria previously reported by Carol et al. [22]. In our slightly modified classification, we included the organizational pattern of neutrophil granulocytes and the presence of monocytes.

The other serial sections were immunohistochemically stained for TLR4, NLRP3, caspase 1, IL-1*β*, IL-18, IL-6, IL-6R, and gp130, all in the same order, using antibodies as listed in Supplementary Table 2. The immune stains were performed using Dako system (Agilent Technologies, Santa Clara, California, USA), an automated immunostain system based on the ABC avidin-biotin-peroxidase method. The system includes deparaffinization, antigen retrieval, and incubation with primary and secondary antibodies, respectively. Enzyme-mediated detection with horseradish peroxidase labelled to diaminobenzidine reporter molecules was used for color detection. All sections were counterstained with hematoxylin. Optimal antigen retrieval, antibody concentrations, and incubation times were pretested with positive and negative controls. The immunostained thrombi were then semiquantitatively graded from negative, to a few [1], some [2], and many [3] positive cells, and classified as either immunohistochemical immune-enzyme negative (IH negative) or positive (IH positive).

Furthermore, dominating type of immunopositive cells (neutrophils and/or monocytes), localization of staining within the cells (membranous, cytoplasmic, and/or nuclear), and registration of extracellular stating were recorded. The histopathological judgements were done individually by two pathologists. Interobserver agreement was tested for the morphological classification and staging of the thrombi in HE sections, as well as for the immunohistochemical scoring of antibody signals in the thrombi. The interobserver agreement was substantial with Kappa scores of 0.82 to 0.86 [23].

### 2.3. Statistical Analyses

Data are presented as mean ± SD, median (25th, 75th percentile), or numbers (%) as appropriate. Nonparametric tests were used, as most variables were skewed distributed. Spearman rho was used for correlation analyses and Mann–Whitney *U*-test for comparing groups. Missing values were excluded from the analyses. *p* values ≤ 0.05 were considered statistically significant. The analyses were performed using STATA IC/15.1.

## 3. Results

### 3.1. Baseline Characteristics

The baseline characteristics of the 33 patients analyzed are shown in Table 1. Median age was 58 years, 91% were male, 49% were current smokers, and 12% had diabetes. Median time from the start of symptoms to PCI was 152 min, whereas median peak troponin T was 3434 *μ*g/L. The culprit lesion was located in the left anterior descending artery (LAD) in about 50% of the patients.

### 3.2. Gene Expression Analyses and Circulating Levels

Of the 33 aspirated thrombi, 30 were successfully analyzed for gene expression. Analyses of gene expression in circulating leukocytes could be done for all patients (*n* = 33) at time of PCI, whereas the next day, three were missing (*n* = 30) for NLRP3, caspase 1, IL-1*β*, IL-18, IL-6R, and gp130; 4 were missing (*n* = 29) for IL-6; and 5 were missing (*n* = 28) for TLR4. All circulating markers were successfully analyzed at the time of PCI (*n* = 33), whereas the next day, one was missing for all markers (*n* = 32).

**Figure 1:**
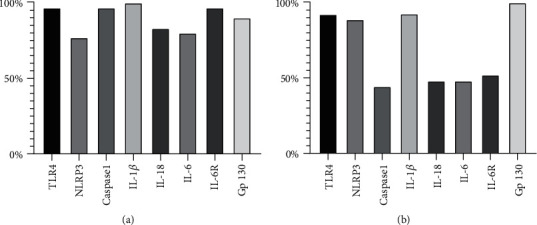
Genes expressed in thrombi. Percentage of thrombi with the different inflammasome-related marker genes expressed ((a), *n* = 30) and found by immunohistochemistry staining ((b), *n* = 27). Abbreviations: TLR4: Toll-like receptor 4; NLRP3: Nod-like receptor protein 3; IL-1*β*: Interleukin 1*β*; IL-18: Interleukin 18; IL-6: Interleukin 6; IL-6R: Interleukin 6 receptor; Gp130: Glycoprotein 130.

We found genes coding for the eight different markers to be present in 76-100% of the thrombi that could be successfully analyzed (Figure 1(a)). Genes encoding IL-1*β* were found in 100%. In circulating leukocytes, all markers were expressed in 100% of successful samples both at the time of PCI and the next day. Levels of gene expression (RQs) in the aspirated thrombi and in circulating leukocytes at the time of aspiration and the next morning (day 1), as well as serum levels at the same time points, are shown in Supplementary Table 1.

We found no significant correlations between genes expressed in thrombi or leukocytes and circulating levels of the corresponding markers at either time points (Supplementary Table 4).

#### 3.2.1. Associations with Myocardial Injury (Table 2)

**Figure 2:**
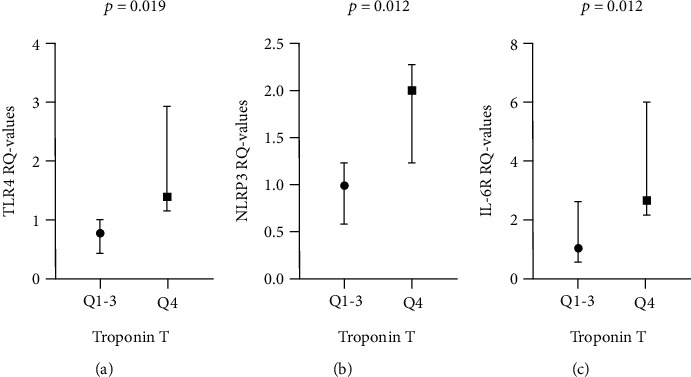
Genes in thrombi related to troponin T. Expression of TLR4 (a) (*n* = 29), NLRP3 (b) (*n* = 23), and IL-6R (c) (*n* = 29) related to troponin T levels dichotomized between quartiles (Qs) 1-3 and Q4 (Wilcoxon rank-sum test). Dots representing medians, vertical lines between 25^th^ and 75^th^ percentiles. TLR4: Toll-like receptor 4; NLRP3: Nod-like receptor protein 3; IL-6R: Interleukin 6 receptor.

The gene expression of TLR4 and NLRP3 in thrombi correlated significantly to peak troponin T (*p* = 0.013 and *p* = 0.024, respectively). When dividing peak troponin T values into quartiles (Qs), TLR4 mRNA in Q4 was 1.8-fold higher (*p* = 0.019) compared to Q1-3, whereas NLRP3 mRNA in Q4 was 2.0-fold higher (*p* = 0.012) compared to Q1-3 (Figures 2(a) and 2(b)). In circulating leukocytes at the time of PCI, the expression of TLR4 and NLRP3 correlated significantly to peak troponin T (*p* = 0.011, *p* = 0.015, respectively), as did also IL-1*β* (*p* = 0.023). A tendency was also observed for Caspase 1 and IL-18, however, not statistically significant. Similar correlations were seen in the samples drawn the next morning (data not shown).

In thrombi, IL-6R expression correlated significantly to peak troponin T (*p* = 0.019), with a 2.5-fold higher median level in Q4 vs. Q1-3 of troponin T (*p* = 0.017) (Figure 2(c)), whereas a significant inverse correlation was observed for gp130 mRNA (*p* = 0.050). In circulating leukocytes, IL-6 expression correlated significantly inverse to peak troponin T (*p* = 0.015). None of the circulating markers, neither at the time of PCI nor the next morning, showed significant correlations to peak troponin T.

Due to the small sample size, any adjustments have not been performed. However, when grouping data according to smoking status and hypertension, no differences could be found comparing Q4 of troponin T vs. Q1-Q3. This comparison could not be done with respect to sex, as there were no female patients in Q4 of troponin T. There was also no significant difference in age when comparing Q4 of troponin T vs. Q1-Q3. Comparing results between patients with diabetes mellitus (*n* = 4) vs. patients without showed no significant differences, and also, when excluding patients with diabetes from the analyses, similar results were obtained. This was also the case when comparing patients on statins (*n* = 6) vs. not on statins and when excluding patients on statins from the analyses.

#### 3.2.2. Associations with Time from Symptom Onset to PCI

As shown in Table 3, there were no significant correlations between genes expressed in the thrombi and time from symptom to PCI. Also, when dividing time at the median (152 min) to explore any cut-off level, no associations with time were observed. In circulating leukocytes at the time of PCI, genes for IL-6 correlated inversely to time (*r* = −0.385, *p* = 0.027), whereas circulating markers did not correlate significantly (Table 3). In samples drawn the next morning (day 1) (data not shown), there were no significant correlations between time to PCI and genes in circulating leukocytes, whereas for circulating markers significant positive correlation for gp130 (*r* = 0.521, *p* = 0.002) was observed (data not shown).

#### 3.2.3. Changes in Levels of Circulating Markers and Gene Expression in Circulating Leukocytes from Time of PCI to the Next Morning (Day 1)

**Figure 3:**
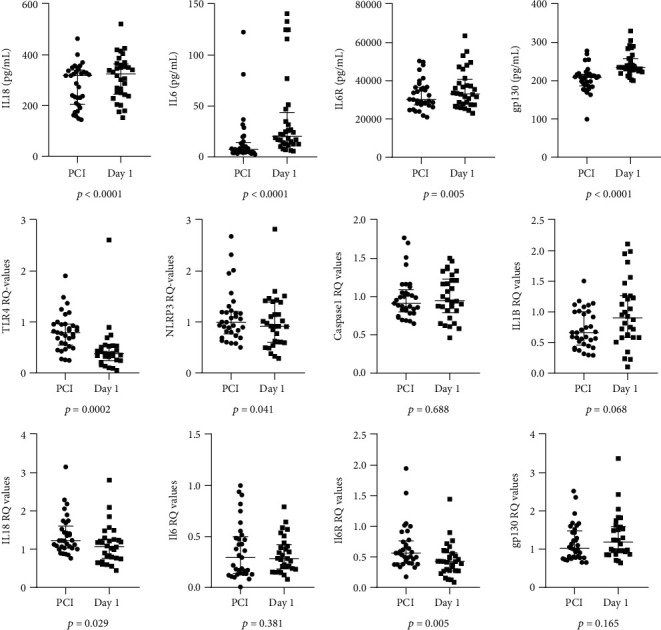
Time of PCI vs. day 1. The first row of graphs shows changes from time of PCI to day 1 in circulating markers (*n* = 32). The second and third row show changes in genes expressed in circulating leukocytes (*n* = 30 for NLRP3, Caspase 1, IL-1*β*, IL-18, IL-6R, and gp130. *n* = 29 for IL-6. *n* = 28 for TLR4) (Wilcoxon rank-sum test). Dots represent individual data. Horizontal lines are drawn at medians, and 25^th^ and 75^th^ percentiles. Abbreviations: TLR4: Toll-like receptor 4; NLRP3: Nod-like receptor protein 3; IL-1*β*: Interleukin 1*β*; IL-18: Interleukin 18; IL-6: Interleukin 6; IL-6R: Interleukin 6 receptor; Gp130: Glycoprotein 130.

As shown in Figure 3, all of the circulating markers were significantly higher the next day compared to the time of PCI (IL-18, IL-6, and gp130 with *p* < 0.0001, IL-6R with *p* = 0.005). mRNA levels of TLR4, NLRP3, IL-18, and IL-6R were significantly lower on the next day (*p* < 0.0001, *p* = 0.041, *p* = 0,029, and *p* = 0.005, respectively), whereas mRNA encoding IL-1*β*, gp130, Caspase 1, and IL6 did not change significantly.

### 3.3. Histology and Immunohistochemistry

Of the 33 aspirated thrombi, 27 contained sufficient material for morphological studies and immunohistochemical analyses.

#### 3.3.1. Thrombus Age

Five (18.5%) of the thrombi were categorized as stage 1 (<1 day old), six (22.2%) as stage 2 (1-5 days old), and two (7.4%) as stage 1+ (1 day + old). 14 of the thrombi (51.9%) showed histological characteristics of both stage 1 and stage 2 and were classified as stage 1 + 2, indicating a process of repeated episodes with fresh bleeding and new thrombus expansion within a lytic thrombus. None of the retrieved thrombi were morphologically organized (stage 3) according to the classification scheme (Supplementary Table 2).

We found no coherent statistically significant differences in the levels of thrombus genes or levels of genes in circulating leukocytes between the different histological stages of thrombus age (Supplementary Table 5). For circulating markers, we found significantly higher levels of IL-6R in the patients with morphologically older thrombi when comparing stage 1 vs. stage 2 (*p* = 0.028) and stage 1 vs. all other stages (*p* = 0.035), but not when comparing stage 2 vs. other stages.

#### 3.3.2. Immunohistochemistry Analyses

Immunohistochemistry staining of the different markers was found in 44% to 100% of the thrombi, as shown in Figure 1(b).

**Figure 4:**
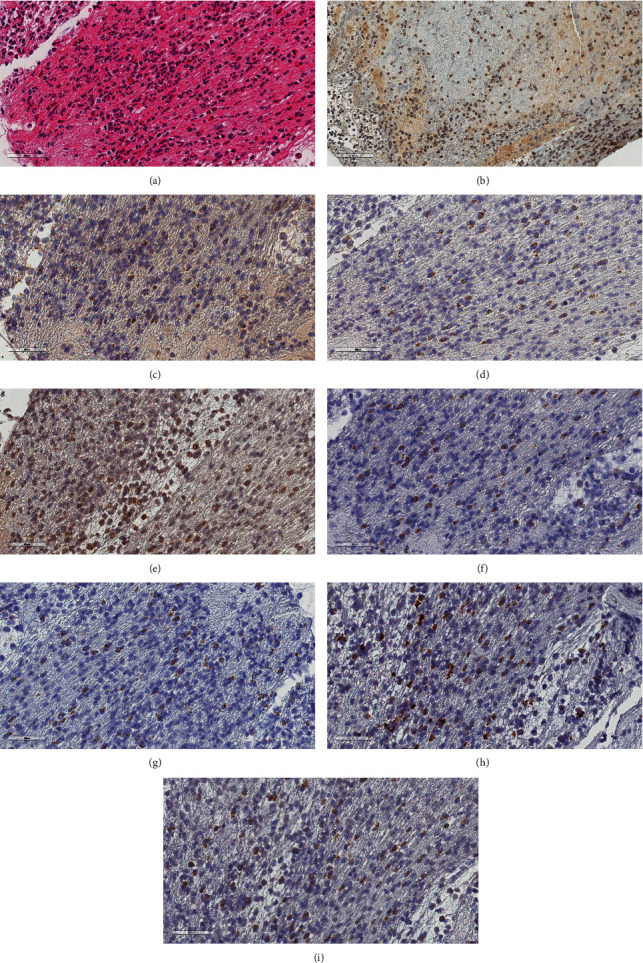
Immunohistochemistry staining in thrombi. Histology and immunohistochemistry (brown signal) of thrombus. Initial magnification × 40, but pictures downsized afterwards. Scale bars indicate 50 micrometers. Histologic age 1 + 2 (Supplementary Table 1). The panels show the following stainings: HE (a), visualizing fibrin organization and varying infiltration of granulocytes and monocytes. TLR4 (b), present in cytoplasm and cell membrane of monocytes and granulocytes. NLRP3 (c), present in cytoplasm of monocytes. Caspase-1 (d), present in nucleus and cytoplasm of monocytes and granulocytes. IL-1ß (e), present in cytoplasm of monocytes and some granulocytes. IL-18 (f), present in cytoplasm of monocytes and granulocytes. IL-6 (g), present in cytoplasm of monocytes. IL-6R (h), present in cytoplasm of monocytes. Gp130 (i), present in nuclear membrane and cytoplasm of monocytes and granulocytes. Abbreviations: HE: hematoxylin and eosin; TLR4: Toll-like receptor 4; NLRP3: Nod-like receptor protein 3; IL-1*β*: Interleukin 1*β*; IL-18: Interleukin 18; IL-6: Interleukin 6; IL-6R: Interleukin 6 receptor; Gp130 = Glycoprotein 130.

Localization by immunohistochemistry staining showed all markers to be present in the cytoplasm of monocytes (Supplementary Table 6). TLR4, IL-1*β*, IL-18, Caspase 1, and gp130 could also be seen in neutrophil granulocytes. In addition to localization in cytosol, TLR4 was seen in the cellular membrane, whereas Caspase 1 and gp130 also could be found in the nuclear membrane. Pictures (a)–(e) in Figure 4 show typical immunohistochemistry staining of the eight different markers, as well as one HE section.

## 4. Discussion

The main finding in our study is that inflammasome-related markers are genetically upregulated in coronary thrombi and circulating leukocytes in patients with STEMI. In thrombi, we found that genes encoding TLR4, NLRP3, and IL-6R were increasingly expressed with increased myocardial damage measured by troponin T. In circulating leukocytes genes encoding TLR4, NLRP3, and IL-1*β* showed positive associations with troponin T.

The Cantos trial [14] showed clinical effect of blocking IL-1*β* in patients with previous myocardial infarction at least 30 days prior to inclusion. Our data of genetic upregulation in thrombi suggest that the inflammasome-related markers are active components already at the time of thrombus formation, indicating that anti-inflammatory treatment targeting the inflammasome signaling axis could be of benefit in the early phase of treating STEMI patients. This has been investigated in the recent ASSAIL-MI trial [24], where a single dose of the IL-6R antagonist tocilizumab was given to patients with STEMI prior to PCI, showing significantly better myocardial salvation index by cardiac MRI in the treated patients vs. placebo [25].

Some of the strongest correlations between peak troponin T and the genetic expression in thrombi in our study were for TLR4 and NLRP3, which both are markers upstream in the inflammasome signaling axis. This can be interpreted as an argument for moving upstream also when medically targeting inflammation in the setting of myocardial infarction. NLRP3 inhibitors have shown promising results in animal models of myocardial infarction [26, 27]. Oral NLRP3 inhibitors have been developed [28] and are tested on patients with gout [29].

Genetic expression of the inflammasome-related markers in circulating leukocytes at the time of PCI was also associated with peak troponin T, especially for genes encoding TLR4, NLRP3, and IL-1*β*, indicating a more generalized upregulation of the genes with myocardial injury. When trying out anti-inflammatory targets in patients with coronary heart disease, it seems that finding the right patients, those with residual inflammatory risk [30], who potentially will benefit the most, is of great importance. In the CANTOS study, they used high-sensitivity C-reactive protein (hsCRP) to identify such patients with hsCRP > 2 mg/L as one of the inclusion criteria [31]. In a later subgroup analysis, they found that the patients on active treatment with the greatest reduction in hsCRP had a 31% reduction in all-cause mortality [32], vs. no significant difference in all-cause mortality for the whole study population. This shows a great heterogenicity in response to treatment, also after selecting patients according to elevated hsCRP. Thus, our findings of genetic upregulation in circulating leukocytes at end of PCI being related to peak troponin T is interesting. It might be hypothesized that expression levels of these genes in the acute setting of a myocardial infarction can help identify patients that will benefit the most from targeting the inflammasome signaling axis. This however needs further elucidation.

Whereas genetic expression of the markers related to the NLRP3 inflammasome, as well as IL-6R, showed a positive association to peak troponin T, mRNA encoding gp130 in thrombi, and IL-6 in circulating leukocytes associated inversely with peak troponin T. This might be explained by the complexity of the IL-6 signaling system, with classic signaling, trans-signaling, soluble form of receptors, and maybe also feedback mechanisms. Circadian cycles of levels of IL-6 in STEMI-patients have been reported [33]. This can affect our results, as all patients in the study were included in daytime. The descriptive design of our study does not provide information to depict these mechanisms further, other than pointing out that the IL-6 signaling system also seems to be actively regulated in the thrombi and circulating leukocytes of these patients. This is underscored by our finding of positive immunohistochemistry enzyme staining of gp130 in the cytoplasm of 100% of the investigated thrombi.

We found that levels of mRNA in circulating leukocytes encoding the inflammasome-related markers were lower the next morning when compared to time of PCI, whereas the circulating markers were higher. This could indicate negative-feedback mechanisms, and hence indicates the importance of measuring mRNA in circulating leukocytes at an early timepoint in the course of a MI for potential identification of patients that will benefit the most from targeting the inflammasome signaling axis.

The immunohistochemistry enzyme analyses in our study also show that the inflammasome-related markers are abundantly present. All of the investigated markers could be found located in the cytoplasm of monocytes in the thrombi. It therefore seems probable that they also play a role in the thrombus itself, and not only in the inflammatory processes taking place in the infarcted myocardium and its border zone after reperfusion. One study has suggested prothrombotic functions of the NLRP3 inflammasome by showing reduced human platelet aggregation response after NLRP3 inhibition in vitro [34]. Several publications in recent years have described the linkage between inflammation, atherosclerosis, and thrombosis in cardiovascular disease [35, 36]. Our findings support the general theory of interplay between innate immune cells, the inflammasome axis, and thrombotic mechanisms actively taking place in human coronary thrombi and may also indicate that the inflammasome-related markers are involved in the formation of intracoronary thrombi.

Statins are suspected to mediate some of their effects through modulating inflammatory responses. Rosuvostatin was shown to attenuate the expression of NLRP3-inflammasome-related markers in circulating monocytes from patients with coronary atherosclerotic disease [37]. In another setting, in patients with ARDS [38], rosuvostatin was shown to associate with elevation in plasma IL-18 levels. In our study, no differences in the thrombi gene expressions in statin users vs. not users were observed, but the number of patients is too small to draw any conclusions.

In STEMI patients, hyperglycemia has been associated with higher thrombus size, different thrombus composition with higher content of macrophages, and higher inflammatory state in their thrombi compared to thrombi from normoglycemic patients [39]. We could not find any significant differences in the investigated genes in the thrombi from patients with diabetes vs. patients without in our study. However, as only 4 patients had diabetes, a larger study is needed to elucidate this aspect.

Our finding does not support any associations between time from symptom onset to PCI and expression of the inflammasome-related markers in thrombi or circulating leukocytes. This is in contrast to a previous report showing some inflammatory markers in coronary thrombi to be associated to ischemic time [10]. In this study, however, median ischemic time was longer (240 vs. 152 min), making it more likely to detect any potential relation to time. Other reasons for the discrepancy can be several. First, time from symptom onset to PCI is a clinical parameter with some inaccuracy. It is well known that many patients have on-and-off symptoms, often over several hours or even days, before an acute worsening sets off contact with healthcare takers. Defining an exact timepoint of onset of symptom can be challenging, if not even impossible. This makes the data uncertain, and the effect of this uncertainty is greater when the number of data is small. Second, intracoronary thrombi might develop stepwise. This is supported by our own findings of different histopathological age-stages within the same thrombi in more than half of the cases, which also matches well with the clinical fact that many patients have on-and-off-symptoms. In these cases, thrombus formation probably is a process exceeding over time until it causes the clinical picture of a STEMI.

We could not find any significant associations between circulating levels of the measured markers and the corresponding genes expressed in thrombi or leukocytes and also no associations to troponin T. This may be due to the low sample size, as circulating IL-6 previously has been shown to associate with troponin T in a larger STEMI population [40].

Another likely reason is that the proteins measured are involved in inflammatory responses in several organs throughout the body, and the amounts reaching the circulation caused by a myocardial infarction are probably too small to have an impact on total levels in the circulation.

### 4.1. Limitations

A major limitation is the small sample size of our study, making adjustments for known risk factors like age, sex, and hypertension difficult. Studying subpopulations can also not be reliably done. Only 3 (9%) were female, which is not representative for a typical cohort of patients with STEMI. This adds to the limitations, as gender differences in troponin levels in patients with AMI [41] and mortality for STEMI [42] have been shown. Patients included in the TASTI study are a selection of STEMI-patients, considered to profit from thrombus aspiration as evaluated by the PCI-operator in charge of the actual procedure. As such, patients included represent a subgroup with an extra tendency for thrombus formation, thus, the generalizability of the study results can be discussed. Nearly all patients were given intravenous heparin and clopidogrel orally before arriving at the hospital, as well as gpIIa/IIIb antagonist during the procedure, before the thrombi were aspirated. This may have affected the thrombi. The markers analyzed are a selection made from a broad range of mediators potentially involved in responses in myocardial ischemic disease, although the selection is based on existing knowledge on the field. Peak troponin T as sole indicator of myocardial injury is also a limitation.

## 5. Conclusions

The inflammasome signaling pathway was actively upregulated in coronary thrombi and in circulating leukocytes in patients with STEMI. Genes encoding TLR4, NLRP3, and IL-6R in the thrombi were increasingly expressed with increased myocardial injury measured by troponin T. In circulating leukocytes genes encoding TLR4, NLRP3, and IL-1*β* associated significantly with troponin T. These results support the strategy of medically targeting this pathway in treating myocardial infarction and contribute to sort out optimal timing and targets for anti-inflammatory treatment.

## Figures and Tables

**Table 1:** Baseline characteristics. Baseline characteristics of the population (*n* = 33). Values are given as absolute numbers (%) or medians (25, 75 percentiles).

Baseline characteristics	
Female gender	3 (9%)
Age (yrs)	58.0 (54.0, 68.0)
Current smokers	16 (49%)
Previous smokers	11 (33%)
BMI (kg/m^2^)	27.7 (23.4, 28.6)
Hypertension	11 (33%)
TDM2	4 (12%)
Previous coronary disease	1 (3%)
Medication before index MI:	
ASA	6 (18%)
Clopidogrel/prasugrel/tikagrelor	2 (6%)
Warfarin	1 (3%)
NOAC	2 (6%)
Beta-blocker	4 (12%)
ACE-I	0 (0%)
AT-II-blocker	5 (15%)
Statins	6 (18%)
Diuretics	4 (12%)
Aldosterone antagonists	0 (0%)
Systolic BP (mmHg)	126.0 (109.0, 144.0)
Diastolic BP (mmHg)	80.0 (70.0, 99.5)
HR (beats/min)	70.0 (65.0, 90)
Ischemic time (min)	152 (122, 343)
CRP (mg/L)	2.71 (1.00, 5.57)
Troponin T after PCI (*μ*/L)	354 (123, 744)
Troponin T peak (*μ*/L)	3434 (1250, 6967)
Culprit lesion:	
LAD	16 (49%)
CX	6 (18%)
RCA	11 (33%)
Retrograde flow	12 (36%)
Three-vessel disease	6 (18%)
In-stent thrombus	1 (3%)

BMI: body mass index; T2DM: type II diabetes mellitus; ASA: acetysalicylic acid; NOAC: novel oral anticoagulant; ACE-I: angiotensin-converting enzyme inhibitor; AT-II-blocker: angiotensin receptor II-blocker; BP: blood pressure; HR: heart rate; CRP: C-reactive protein; PCI: percutan coronary intervention; LAD: left anterior descending artery; CX: circumflex artery; RCA: right coronary artery.

**Table 2:** Relation to peak troponin T. Spearman coefficients of correlations (rho) between the markers measured at the time of PCI and peak troponin T.

	Genes in thrombus		Genes in circ. leuk.		Circulating marker	
	Rho	*p*	Rho	*p*	Rho	*p*
TLR4	0.455	*0.013*	0.438	*0.011*		
NLRP3	0.468	*0.024*	0.420	*0.015*		
Caspase1	0.210	0.273	0.321	0.069		
IL-1*β*	0.199	0.292	0.394	*0.023*		
IL-18	0.159	0.447	0.310	0.079	-0.084	0.641
IL-6	0.101	0.639	-0.422	*0.015*	0.184	0.307
IL-6R	0.434	*0.019*	0.197	0.271	-0.048	0.793
gp130	-0.380	*0.050*	-0.047	0.794	0.071	0.694

*p* ≤ 0.05 italicized as sign of statistical significance.

**Table 3:** Relation to time from symptom to PCI. Spearman coefficients of correlations (rho) between the markers measured at the time of PCI and time from symptom to PCI.

	Genes in thrombus		Genes in circ. leuk.		Circulating marker	
	Rho	*p*	Rho	*p*	Rho	*p*
TLR4	0.079	0.684	-0.271	0.127		
NLRP3	0.031	0.890	-0.165	0.360		
Caspase 1	-0.032	0.868	-0.126	0.486		
IL-1*β*	0.137	0.469	-0.147	0.414		
IL-18	-0.265	0.200	-0.068	0.708	0.259	0.146
IL-6	0.127	0.553	-0.385	*0.027*	0.232	0.195
IL-6R	0.142	0.462	-0.265	0.136	-0.039	0.830
gp130	-0.128	0.526	-0.129	0.476	0.291	0.101

*p* ≤ 0.05 italicized as a sign of statistical significance.

## Data Availability

The data that support the findings of this study are available from the corresponding author upon reasonable request.

## Supplementary Materials

Supplementary Materials Supplementary Table 1: gene expression levels. Gene expression levels (RQ) in the aspirated thrombi, circulating leukocytes, and serum levels. Supplementary Table 2: histologic thrombus age stages. Estimated age and histologic characteristics of the different thrombus age stages. Supplementary Table 3: antibodies. Listing of antibodies used for immunohistochemistry analyses. Supplementary Table 4: circulating markers towards genes. Correlations between circulating markers and corresponding genes expressed in thrombi and in circulating leukocytes. Supplementary Table 5: differences between histologic age stages. Data grouped according to histologic age stages. Supplementary Table 6: localization by immunohistochemistry staining. Cell types, and localization within the cells, of the different markers visualized with immunohistochemistry staining. Supplementary Table 7: STROBE Statement Checklist.
