# Supplementary material for: The Inflammasome Signaling Pathway Is Actively Regulated and Related to Myocardial Damage in Coronary Thrombi from Patients with STEMI
Source: Mediators Inflamm. 2021 May 27;2021:5525917. doi: 10.1155/2021/5525917 (PMC8178014; doi:10.1155/2021/5525917)
Supplement: Supplementary Materials — Supplementary Table 1: gene expression levels. Gene expression levels (RQ) in the aspirated thrombi, circulating leukocytes, and serum levels. Supplementary Table 2: histologic thrombus age stages. Estimated age and histologic characteristics of the different thrombus age stages. Supplementary Table 3: antibodies. Listing of antibodies used for immunohistochemistry analyses. Supplementary Table 4: circulating markers towards genes. Correlations between circulating markers and corresponding genes expressed in thrombi and in circulating leukocytes. Supplementary Table 5: differences between histologic age stages. Data grouped according to histologic age stages. Supplementary Table 6: localization by immunohistochemistry staining. Cell types, and localization within the cells, of the different markers visualized with immunohistochemistry staining. Supplementary Table 7: STROBE Statement Checklist. [file 5525917.f1.docx]

**Supplementary materials**

***Supplementary Table 1. Gene expression levels.***

Gene expression levels (RQ) in the aspirated thrombi, circulating leukocytes and serum levels. Medians (25, 75 percentiles) are given.

|  | RQ values  Thrombi | RQ values  Leukocytes  Aspiration time | RQ values  Leukocytes  Day1 | Circulating levels (pg/mL)  Aspiration time | Circulating levels (pg/mL)  Day 1 |
| --- | --- | --- | --- | --- | --- |
| TLR4 | 0.90 (0.52, 1.48) | 0.79 (0.57, 0.96) | 0.38 (0.25, 0.53) |  |  |
| NLRP3 | 1.18 (0.62, 1.79) | 1.00 (0.80, 1.19) | 0.92 (0.61, 1.39) | - | - |
| Caspase1 | 1.00 (0.60, 1.72) | 0.91 (0.82, 1.05) | 0.95 (0.82, 1.21) | - | - |
| IL-1β | 3.44 (1.00, 5.68) | 0.66 (0.47, 1.00) | 0.90 (0.58, 1.26) | - | - |
| IL-18 | 1.51 (0.91, 2.66) | 1.23 (1.03, 1.53) | 1.07 (0.77, 1.26) | 318 (209, 337) | 325 (247, 363) |
| IL-6 | 0.14 (0.04, 0.45) | 0.29 (0.14, 0.48) | 0.28 (0.19, 0.41) | 7.40 (4.61, 13.30) | 20.30 (12.69, 40.83) |
| IL-6R | 1.95 (0.93, 2.77) | 0.56 (0.40, 0.75) | 0.43 (0.27, 0.56) | 30148 (27527, 36567) | 33199 (27741, 40083) |
| Gp130 | 0.61 (0.48, 1.03) | 1.03 (0.78, 1.44) | 1.18 (0.93, 1.58) | 209 (188, 215) | 235 (227, 257) |

***Supplementary Table 2. Histologic thrombus age stages.***

Estimated age and histologic characteristics of the different thrombus age stages.

| Thrombus stage |  | Estimated age | Histologic characteristics |
| --- | --- | --- | --- |
| Stage 1 | Fresh | < 1 day | Platelet aggregates, erythrocytes/fresh bleeding, intact granulocytes in focal areas, fibrin of varying organization |
| Stage 1+ |  | 1 day + | Platelet aggregates, erythrocytes/fresh bleeding, intact granulocytes in focal areas and fibrin of varying organization, small areas with disintegration of granulocytes |
| Stage 2 | Lytic | 1-5 days | Areas of colliquative (liquefactive) necrosis and diffuse spreading of granulocyte with karyorrhexis and necrosis, increasing number of monocytes. |
| Stage 3 | Organized | > 5 days | Presence of smooth muscle cells, homogeneous or hyaline fibrin. Depositions of fibroblasts and endothelial cells. |

***Supplementary Table 3. Antibodies.***

Listing of antibodies used for immunohistochemistry analyzes.

| **Antibody** | **Source** | **Dilution** |
| --- | --- | --- |
| TLR4 | ab13867, Abcam,  Polyclonal rabbit | 1/100 |
| NLRP3 | ab214185, Abcam,  Polyclonal rabbit | 1/200 |
| Caspase1 | sc-56036, Santa Cruz Biotechnology,  Monoclonal mouse | 1/2000 |
| IL-1ß | ab2105, Abcam, Polyclonal rabbit | 1/200 |
| IL-18 | HPA003980, Biocompare,  Polyclonal rabbit | 1/1000 |
| IL-6 | ab6672, Abcam,  Polyclonal rabbit | 1/800 |
| IL-6R | ab128008, Abcam,  Polyclonal rabbit | 1/1000 |
| gp130 | Anti-CD130 (gp130) antibody ab202850, Abcam, Polyclonal rabbit | 1/1000 |

***Supplementary Table 4 Circulating markers towards genes.***

Correlations between circulating markers and corresponding genes expressed in thrombi and in circulating leukocytes (Spearmans rho).

|  | **GENES IN THROMBUS** | |  |  | **GENES IN CIRC LEUK** | | |
| --- | --- | --- | --- | --- | --- | --- | --- |
| Circulating levels |  |  |  | Day 1 |  | Day 2 |  |
|  | Rho | p |  | Rho | P | Rho | p |
| IL-18 Day 1 | -0.172 | 0.412 |  | 0.027 | 0.883 | -0.046 | 0.808 |
| Day 2 | -0.279 | 0.187 |  | 0.017 | 0.927 | -0.165 | 0.384 |
| IL-6 Day 1 | -0.159 | 0.459 |  | -0.183 | 0.306 | -0.169 | 0.382 |
| Day 2 | -0.181 | 0.409 |  | -0.100 | 0.587 | -0.104 | 0.590 |
| IL-6R Day 1 | 0.205 | 0.285 |  | 0.103 | 0.570 | -0.094 | 0.623 |
| Day 2 | 0.334 | 0.083 |  | -0.149 | 0.415 | -0.163 | 0.391 |
| gp130 Day1 | -0.047 | 0.816 |  | 0.057 | 0.752 | 0.125 | 0.510 |
| Day 2 | 0.001 | 0.996 |  | -0.027 | 0.883 | 0.160 | 0.400 |

***Supplementary Table 5. Differences between histologic age stages.***

Data grouped according to histologic age stages. A: 1 vs other; B: 2 vs other; C: 1 vs 2. Groups compared by Mann Whitney U test. Data are givens as medians (25^th^, 75^th^-percentiles).

5A

|  |  | Day 1 |  |  | Day 2 |  |  |
| --- | --- | --- | --- | --- | --- | --- | --- |
|  |  | Stage 1 | Other | p | Stage 1 | Other | p |
| **Genes in thrombus** | |  |  |  |  |  |  |
| TLR4 |  | 1.55 (0.43, 2.88) | 0.90 (0.61,1.24) | 0.416 |  |  |  |
| NLRP3 |  | 1.01 (0.21, 1.17) | 1.23 (0.96, 1.79) | 0.186 |  |  |  |
| Caspase1 |  | 0.92 (0.43, 1.48) | 1.00 (0.70, 1.59) | 0.712 |  |  |  |
| IL-1β |  | 3.93 (1.92, 6.53) | 3.29 (0.81, 5.40) | 0.380 |  |  |  |
| IL-18 |  | 4.37 (0.73, 13.58) | 1.78 (0.91, 2.66) | 0.734 |  |  |  |
| IL-6 |  | 0.02 (0.01, 0.06) | 0.16 (0.06, 0.47) | 0.0502 |  |  |  |
| IL-6R |  | 2.66 (1.31, 6.76) | 1.95 (1.00, 2.73) | 0.313 |  |  |  |
| gp130 |  | 0.78 (0.15, 0.95) | 0.61 (0.48, 1.03) | 0.458 |  |  |  |
|  |  |  |  |  |  |  |  |
| **Markers in circulation** | |  |  |  |  |  |  |
| IL18 |  | 287 (209, 331) | 279 (201,343) | 0.851 | 287 (228, 306) | 332 (250, 370) | 0.255 |
| IL-6 |  | 8.50 (4.67, 20.72) | 8.17 (4.61, 13.3) | 0.662 | 24.13 (15.12, 77.00) | 22.27 (13.89, 46.89) | 0.745 |
| IL-6R |  | 29600 (28137, 30148) | 31979 (27161, 36751) | 0.417 | 26673 (26246, 31612) | 33627 (28685, 46197) | **0.035** |
| gp130 |  | 223 (193, 255) | 208 (184, 214) | 0.212 | 284 (232,286) | 235 (228,257) | 0.416 |
|  |  |  |  |  |  |  |  |
| **Genes in leukocytes** | |  |  |  |  |  |  |
| TLR4 |  | 0.84 (0.68, 0.96) | 0.76 (0.52, 0.93) | 0.803 | 0.42 (0.24, 0.51) | 0.38 (0.32, 0.54) | 0.611 |
| NLRP3 |  | 1.08 (1.00, 1.19) | 0.97 (0.81, 1.22) | 0.803 | 0.92 (0.61, 1.15) | 1.01 (0.61, 1.43) | 0.414 |
| Caspase1 |  | 1.00 (0.85, 1.05) | 0.90 (0.83, 1.03) | 0.618 | 0.94 (0.88, 1.07) | 1.11 (0.82, 1.34) | 0.546 |
| IL-1β |  | 1.00 (0.59, 1.01) | 0.59 (0.41, 0.92) | 0.417 | 1.05 (0.63, 1.21) | 1.00 (0.58, 1.47) | 0.804 |
| IL-18 |  | 1.53 (1.00, 1.74) | 1.22 (1.07, 1.44) | 0.756 | 0.79 (0.77, 1.50) | 1.06 (0.74, 1.26) | 0.804 |
| IL-6 |  | 0.29 (0.13, 0.82) | 0.20 (0.14, 0.43) | 0.803 | 0.31 (0.21, 0.57) | 0.22 (0.18, 0.41) | 0.516 |
| IL-6R |  | 0.56 (0.48, 0.56) | 0.52 (0.40, 0.70) | 0.852 | 0.50 (0.23, 0.67) | 0.42 (0.28, 0.56) | 0.915 |
| gp130 |  | 1.23 (1.00, 1.29) | 0.98 (0.78, 1.44) | 0.349 | 1.49 (1.24, 1.53) | 1.14 (0.86, 1.83) | 0.456 |

Thrombustsage 1, n = 5. Thrombusstage Other (1+, 1+2, 2), n = 22. p≤0.05 bolded as sign of statistical significance.

5B.

|  |  | Day 1 |  |  | Day 2 |  |  |
| --- | --- | --- | --- | --- | --- | --- | --- |
|  |  | Other stages | Stage 2 | p | Other stages | Stage 2 | p |
| **Genes in thrombus** | |  |  |  |  |  |  |
| TLR4 |  | 1.00 (0.62, 1.55) | 0.83 (0.52, 1.37) | 0.770 |  |  |  |
| NLRP3 |  | 1.24 (0.98, 1.99) | 0.89 (0.58, 1.20) | 0.186 |  |  |  |
| Caspase1 |  | 1.01 (0.65, 1.77) | 0.96 (0.64, 1.20) | 0.497 |  |  |  |
| IL-1β |  | 3.59 (1.33, 5.68) | 2.33 (0.74, 5.40) | 0.649 |  |  |  |
| IL-18 |  | 1.78 (0.93, 2.66) | 4.54 (0.70, 17.62) | 0.734 |  |  |  |
| IL-6 |  | 0.10 (0.03, 0.43) | 0.16 (0.05, 0.38) | 0.845 |  |  |  |
| IL-6R |  | 2.18 (1.07, 2.73) | 1.00 (0.86, 2.77) | 0.626 |  |  |  |
| gp130 |  | 0.61 (0.48, 1.03) | 0.96 (0.47, 1.03) | 0.644 |  |  |  |
| **Markers in circulation** | |  |  |  |  |  |  |
| IL-18 |  | 287 (209, 331) | 278 (158, 357) | 0.884 | 306 (243, 360) | 341 (253, 425) | 0.580 |
| IL-6 |  | 8.28 (4.67, 15.09) | 9.10 (4.20, 13.30) | 0.771 | 21.78 (13.89, 51.26) | 26.67 (25.98, 31.70) | 0.495 |
| IL-6R |  | 29600 (26246, 35524) | 34974 (27527, 36751) | 0.351 | 31673 (27527, 41382) | 36751 (33443, 41382) | 0.380 |
| gp130 |  | 208 (188, 223) | 210 (178, 212) | 0.502 | 235 (223, 267) | 238 (230, 240) | 0.897 |
|  |  |  |  |  |  |  |  |
| **Genes in leukocytes** | |  |  |  |  |  |  |
| TLR4 |  | 0.79 (0.59,0.96) | 0.61 (0.49, 0.89) | 0.294 | 0.40 (0.24, 0.53) | 0.34 (0.29, 0.54) | 0.865 |
| NLRP3 |  | 1.00 (0.89, 1.22) | 0.87 (0.63, 1.11) | 0.180 | 1.02 (0.61, 1.42) | 0.92 (0.61, 1.01) | 0.500 |
| Caspase1 |  | 0.96 (0.84, 1.05) | 0.99 (0.78, 1.16) | 0.466 | 1.07 (0.87, 1.33) | 0.82 (0.72, 1.11) | 0.500 |
| IL-1β |  | 0.66 (0.41, 1.01) | 0.58 (0.52, 0.66) | 0.726 | 1.05 (0.58, 1.57) | 0.82 (0.72, 1.13) | 0.500 |
| IL-18 |  | 1.23 (1.07, 1.69) | 1.18 (1.07, 1.37) | 0.726 | 1.06 (0.79, 1.48) | 0.74 (0.67, 1.23) | 0.414 |
| IL-6 |  | 0.22 (0.14, 0.43) | 0.23 (0.12, 0.47) | 0.771 | 0.27 (0.18, 0.38) | 0.21 (0.21, 0.41) | 0.823 |
| IL-6R |  | 0.54 (0.44, 0.67) | 0.55 (0.38, 0.78) | 0.954 | 0.43 (0.27, 0.67) | 0.39 (0.30, 0.43) | 0.414 |
| gp130 |  | 1.00 (0.78, 1.29) | 1.33 (0.91, 1.60) | 0.382 | 1.24 (0.87, 1.58) | 1.00 (0.96, 1.75) | 0.859 |

Thrombusstage 2, n = 6. Thrombusstage Other (1, 1+, 1+2), n = 21

5C.

|  |  | Day 1 |  |  | Day 2 |  |  |
| --- | --- | --- | --- | --- | --- | --- | --- |
|  |  | Stage 1 | Stage 2 | p | Stage 1 | Stage 2 | p |
| **Genes in thrombus** | |  |  |  |  |  |  |
| TLR4 |  | 1.55 (0.43, 2.88) | 0.83 (0.52, 1.37 | 0.602 |  |  |  |
| NLRP3 |  | 1.01 (0.21, 1.17) | 0.89 (0.58, 1.20) | 0.480 |  |  |  |
| Caspase1 |  | 0.92 (0.43, 1.48) | 0.96 (0.64, 1.20) | 0.807 |  |  |  |
| IL-1β |  | 3.93 (1.92, 6.53) | 2.33 (0.74, 5.40) | 0.465 |  |  |  |
| IL-18 |  | 4.37 (0.73, 13.58) | 4.54 (0.70, 17.62) | 1.000 |  |  |  |
| IL-6 |  | 0.02 (0.01, 0.06) | 0.16 (0.05, 0.38) | 0.221 |  |  |  |
| IL-6R |  | 2.66 (1.31, 6.76) | 1.00 (0.86, 2.77) | 0.347 |  |  |  |
| gp130 |  | 0.78 (0.15, 0.95) | 0.96 (0.47, 1.03) | 0.297 |  |  |  |
|  |  |  |  |  |  |  |  |
| **Markers in circulation** | |  |  |  |  |  |  |
| IL-18 |  | 287 (209, 331) | 278 (158, 357) | 1 | 287 (228, 306) | 341 (253, 415) | 0.347 |
| IL-6 |  | 8.50 (4.67, 20.72) | 9.10 (4.20, 13.30) | 0.465 | 24.13 (15.12, 77.00) | 26.67 (25.98, 31.70) | 0.754 |
| IL-6R |  | 29600 (28137, 30148) | 34974 (27527, 36751) | 0.201 | 26673 (28246, 31612) | 36751 (33443, 37243) | **0.028** |
| gp130 |  | 223 (193, 255) | 210 (178, 212) | 0.201 | 284 (232, 286) | 238 (230, 240) | 0.602 |
|  |  |  |  |  |  |  |  |
| **Genes in leukocytes** | |  |  |  |  |  |  |
| TLR4 |  | 0.84 (0.68, 0.96) | 0.61 (0.49, 0.89) | 0.465 | 0.42 (0.24, 0.51) | 0.34 (0.29, 0.54) | 0.807 |
| NLRP3 |  | 1.08 (1.00, 1.19) | 0.87 (0.63, 1.11) | 0.361 | 0.92 (0.61, 1.15) | 0.92 (0.61, 1.01) | 0.754 |
| Caspase1 |  | 1.00 (0.85, 1.05) | 0.89 (0.68, 0.99) | 0.465 | 0.94 (0.88, 1.07) | 0.82 (0.72, 1.11) | 0.917 |
| IL-1β |  | 1.00 (0.59, 1.01) | 0.58 (0.52, 0.66) | 0.273 | 1.05 (0.63, 1.21) | 0.82 (0.72, 1.13) | 0.917 |
| IL-18 |  | 1.53 (1.00, 1.74) | 1.18 (1.07, 1.37) | 0.715 | 0.79 (0.77, 1,50) | 0.74 (0.67, 1.23) | 0.465 |
| IL-6 |  | 0.29 (0.13, 0.82) | 0.23 (0.12, 0.47) | 0.715 | 0.31 (0.21, 0.57) | 0.21 (0.21, 0.41) | 0.807 |
| IL-6R |  | 0.56 (0.48, 0.56) | 0.55 (0.38, 0.78) | 0.855 | 0.50 (0.23, 0.67) | 0.39 (0.30, 0.43) | 0.602 |
| gp130 |  | 1.23 (1.00, 1.29) | 1.33 (1.60, 0.91) | 1.000 | 1.49 (1.24, 1.53) | 1.00 (0.96, 1.75) | 0.602 |

Thrombustsage 1, n = 5. Thrombusstage 2, n = 6. p≤0.05 bolded as sign of statistical significance.

***Supplementary Table 6. Localization by immunohistochemistry staining.***

Cell types, and localization within the cells, of the different markers visualized with immunohistochemistry staining.

| **Immune marker** | **Cell type** | **Localization** |
| --- | --- | --- |
| TLR4 | Monocytes and granulocytes | Cytoplasmic and cellular membrane |
| NLRP 3 | Monocytes | Cytoplasmic |
| Caspase1 | Monocytes and granulocytes | Cytoplasmic and nuclear membrane |
| IL-1ß | Monocytes and some granulocytes | Cytoplasmic |
| IL-18 | Monocytes and granulocytes | Cytoplasmic |
| IL-6 | Monocytes | Cytoplasmic |
| IL-6R | Monocytes | Cytoplasmic |
| gp130 | Monocytes and granulocytes | Cytoplasmic and nuclear membrane |

***Supplementary Table 7. STROBE Statement Checklist***

STROBE Statement—Checklist of items that should be included in reports of ***cross-sectional studies***

|  | Item No | Recommendation | Page No |
| --- | --- | --- | --- |
| **Title and abstract** | 1 | (*a*) Indicate the study’s design with a commonly used term in the title or the abstract | 2 |
|  |  | (*b*) Provide in the abstract an informative and balanced summary of what was done and what was found | 2,3 |
| Introduction | | | |
| Background/rationale | 2 | Explain the scientific background and rationale for the investigation being reported | 4,5 |
| Objectives | 3 | State specific objectives, including any prespecified hypotheses | 5,6 |
| Methods | | | |
| Study design | 4 | Present key elements of study design early in the paper | 6,7 |
| Setting | 5 | Describe the setting, locations, and relevant dates, including periods of recruitment, exposure, follow-up, and data collection | 6,7 |
| Participants | 6 | (*a*) Give the eligibility criteria, and the sources and methods of selection of participants | 6,7 |
| Variables | 7 | Clearly define all outcomes, exposures, predictors, potential confounders, and effect modifiers. Give diagnostic criteria, if applicable | N/A |
| Data sources/ measurement | 8* | For each variable of interest, give sources of data and details of methods of assessment (measurement). Describe comparability of assessment methods if there is more than one group | 7,8,9,10 |
| Bias | 9 | Describe any efforts to address potential sources of bias | 12 |
| Study size | 10 | Explain how the study size was arrived at | N/A |
| Quantitative variables | 11 | Explain how quantitative variables were handled in the analyses. If applicable, describe which groupings were chosen and why | 7,8,9,10 |
| Statistical methods | 12 | (*a*) Describe all statistical methods, including those used to control for confounding | 10 |
|  |  | (*b*) Describe any methods used to examine subgroups and interactions | N/A |
|  |  | (*c*) Explain how missing data were addressed | 10 |
|  |  | (*d*) If applicable, describe analytical methods taking account of sampling strategy | N/A |
|  |  | (*e*) Describe any sensitivity analyses | N/A |
| Results | | | |
| Participants | 13* | (a) Report numbers of individuals at each stage of study—eg numbers potentially eligible, examined for eligibility, confirmed eligible, included in the study, completing follow-up, and analysed | N/A |
|  |  | (b) Give reasons for non-participation at each stage | N/A |
|  |  | (c) Consider use of a flow diagram | N/A |
| Descriptive data | 14* | (a) Give characteristics of study participants (eg demographic, clinical, social) and information on exposures and potential confounders | 10, 26, 27 |
|  |  | (b) Indicate number of participants with missing data for each variable of interest | 11, 13 |
| Outcome data | 15* | Report numbers of outcome events or summary measures | N/A |
| Main results | 16 | (*a*) Give unadjusted estimates and, if applicable, confounder-adjusted estimates and their precision (eg, 95% confidence interval). Make clear which confounders were adjusted for and why they were included | N/A |
|  |  | (*b*) Report category boundaries when continuous variables were categorized | N/A |
|  |  | (*c*) If relevant, consider translating estimates of relative risk into absolute risk for a meaningful time period | N/A |
| Other analyses | 17 | Report other analyses done—eg analyses of subgroups and interactions, and sensitivity analyses | 12 |
| Discussion | | | |
| Key results | 18 | Summarise key results with reference to study objectives | 21 |
| Limitations | 19 | Discuss limitations of the study, taking into account sources of potential bias or imprecision. Discuss both direction and magnitude of any potential bias | 20 |
| Interpretation | 20 | Give a cautious overall interpretation of results considering objectives, limitations, multiplicity of analyses, results from similar studies, and other relevant evidence | 15,16,17,18,19,20,21 |
| Generalisability | 21 | Discuss the generalisability (external validity) of the study results | 20 |
| Other information | | | |
| Funding | 22 | Give the source of funding and the role of the funders for the present study and, if applicable, for the original study on which the present article is based | 22 |
